# Photoactive Hydrogels as Materials for Biological Applications: Preparation of Thermally Stable Photoactive Films

**DOI:** 10.3390/gels11080663

**Published:** 2025-08-20

**Authors:** Oscar G. Marambio, Lidia Álvarez, Héctor Díaz-Chamorro, Julio Sánchez, Rudy Martin-Trasancos, Christian Erick Palavecino, Guadalupe del C. Pizarro

**Affiliations:** 1 Departamento de Química, Facultad de Ciencias Naturales, Matemáticas y Medio Ambiente, Universidad Tecnológica Metropolitana (UTEM), J. P. Alessandri 1242, Santiago 7800002, Chile; omarambi@utem.cl (O.G.M.); lidia.alvarezc@utem.cl (L.Á.); hdiaz@utem.cl (H.D.-C.); 2 Departamento de Química Orgánica, Facultad de Química y de Farmacia, Pontificia Universidad Católica de Chile, Santiago 7820436, Chile; julio.sanchez@uc.cl; 3 Departamento de Ingeniería Mecánica, Facultad de Ingeniería, Universidad de Santiago de Chile (USACH), Av. Bernardo O’Higgins 3363, Santiago 9170002, Chile; rudy.martin@usach.cl; 4 Laboratorio de Microbiología Celular, Centro de Ciencias Médicas Aplicadas, Facultad de Medicina y Ciencias de la Salud, Universidad Central de Chile, Lord Cochrane 418, Santiago 8330546, Chile

**Keywords:** bioinspired material, stimulus-response hydrogel films, biological applications

## Abstract

Hydrogel materials have become an efficient, bioactive, and multifunctional alternative with great potential for biomedical applications. In this work, photoactive films were successfully designed for optical processing, and their photoactivity was tested in photodynamic therapy (PDT), such as antimicrobial patches. The stimulus-response hydrogel films are made of a hydrophilic polymer based on vinyl monomers, specifically 2-hydroxyethyl methacrylate (HEMA) and acrylamide (AAm), in a 1:1 molar ratio, along with the photochromic agent, 3,3-dimethylindolin-6′-nitrobenzoespiropirano (BSP), and a crosslinking agent, N,N’-methylenebisacrylamide (MBA). These hydrogel films were successfully created using the photoinitiator 2-hydroxy-4′-(2-hydroxyethoxy)-2-methylpropiophenone (IRGACURE 2959), MBA, and BSP in different concentrations (0.1, 0.3, and 0.5 mol%), which were later tested in photodynamic therapy (PDT) with the photosensitizer Ru(bpy)_2_^2+^ against *Staphylococcus aureus*. The results showed that, while free Ru(bpy)_2_^2+^ needed concentrations of 4–8 µg/mL to eliminate methicillin-sensitive (MSSA) strains, only partial inactivation was achieved for methicillin-resistant (MRSA) strains. The addition of the hydrogel films with BSP improved their effectiveness, lowering the minimum inhibitory concentration (MIC) to 2 µg/mL to fully inactivate MSSA and MRSA strains. These findings demonstrate that the combined use of hydrogel films containing BSP and Ru(bpy)_2_^2+^ within a hydrogel matrix not only boosts antimicrobial activity but also highlights the potential of these photoactive films as innovative photosensitive antimicrobial coatings. This synergistic effect of ***BSP*** and Ru(bpy)_2_^2+^ indicates that these materials are promising candidates for next-generation antimicrobial coatings and creative photosensitive materials.

## 1. Introduction

Hydrogel materials have become one of the most popular materials depending on their application, such as in contact lenses, oil recovery, pharmaceuticals, agriculture, textiles, and adherent materials [1,2,3,4,5,6,7]. They have emerged as an efficient bioactive multifunctional alternative [8,9,10,11,12]. These hydrogels can be cross-linked via thermal polymerization [13,14], photopolymerization [15,16], enzymatic crosslinking [17], and several other methods [18].

The crosslinking process can be ionic or covalent and can change a macromolecule into solid or gel material by restricting its mobility [19]. Chemically cross-linked hydrogels are not reversible and have higher mechanical properties [19]. The strength of hydrogels can be attributed to different types of interactions, such as hydrogen bonds [20], ionic bonds [21], hydrophobic bonds [22,23], and hydrophilic bonds [24,25].

The physicochemical properties of hydrogels can be easily modified, allowing them to be tailored for various biomedical applications. Hydrogel adhesive patches are widely used for transdermal drug delivery and have also found commercial applications. Beyond transdermal delivery, these hydrogel patches also have applications in cardiac therapy, cancer research, biosensing, and more [26,27,28,29].

Spiropyran is compatible with most polymerization conditions; therefore, it has been used with monomers, such as those used for grafting onto preformed polymer chains. On the other hand, various approaches have been developed for spiropyran-linked polymers. The grafting approach has been employed to functionalize a variety of polymers, including polytetrafluoroethylene (PTFE) [30], polyaniline [31], polyacrylates [32], polysulfones [33], and polyphosphazenes [34,35].

Spiropyran (SP), one of the most renowned hydrophobic photochromic compounds, is photoisomerized to the zwitterionic merocyanine (***MC***) form by UV light irradiation. Merocyanine is then converted back to ***SP*** by visible light irradiation or heating. An essential property of ***MC*** is its ability to form metal ion complexes. The preparation of honeycomb films containing a photochromic ***SP*** moiety and the photopatterning of these films have been previously reported [36,37,38,39].

On the other hand, spiropyran can also be adapted to detect diagnostic biomarkers for various human diseases. In a recently reported work, it was proposed that the bis-spiropyran dyad could serve as a fluorescent probe to monitor intracellular levels of γ-glutamyl-cysteinyl-glycine (GSH) [40]. Abnormal GSH levels are associated with various diseases, including cystic fibrosis, neurodegenerative diseases, and cancer. In a 20% ethanol solution, the spiropyran probe was colorless, and UV irradiation did not alter the uptake, indicating the absence of the MC form. However, the addition of GSH caused a dramatic change in the uptake of the spiropyran in ethanol solution. The specificity of detection was further examined by adding cysteine (Cys) or glutamic acid (Glu), which did not induce changes in uptake. The fluorescence emission of spiropyran was also shifted from 517 nm without GSH to 643 nm in the presence of GSH when excited at the wavelength of maximum absorption. Notably, the same probe showed different behavior upon introducing a nitro substituent instead of a tert-butyl group at position 60 [41]. The new derivative originally existed as a highly fluorescent MC isomer in water, and upon addition of GSH, the SP isomer was formed, resulting in decreased emission. In addition to GSH, spiropyran-modified multi-walled carbon nanotubes (MWNTs) were successfully used to regulate horseradish peroxidase (HRP) activity by light [42]. HRP showed enhanced activity in the presence of MWNT-MC, but MWNT-SP had no effect. By using the increased activity of HRP, a highly sensitive and selective lysozyme detection system was developed [42]. This approach is highly convenient and practical, as it does not require any covalent modification of spiropyran to a target protein, and can therefore be applied to various natural proteins. Furthermore, spiropyran-modified single-walled carbon nanotubes (SWNTs) displayed amplified electrochemical signals, which were used to achieve photochemical and target-modulated electron transfer simultaneously.

Therefore, some factors affect hydrogels’ swelling ratio, such as chemical structure and composition of monomers [43,44], network structure [45], crosslinking ratio, and the specific stimuli or the surrounding medium [46,47]. Swelling and absorption properties of these materials are attributed to the presence of hydrophilic groups in the network [43]. For example, PHEMA has been employed in pharmaceutical and biomaterial applications [48,49]. Moreover, injuries caused by adhesive patches pose a significant threat to full-thickness skin trauma, as their strong adhesion can lead to severe pain and exacerbate the injury upon removal.

Stimuli-responsive hydrogels have become an exciting class of biomaterials for advanced wound healing, offering adaptable and controllable responses to the wound environment. These hydrogels are designed to activate in response to specific stimuli, such as pH, temperature, light, and enzyme activity, enabling precise control of drug delivery, antimicrobial effects, and tissue repair. Composite stimuli-responsive hydrogels, which combine multiple response mechanisms and functions, show promise for addressing the diverse needs of wound healing. However, challenges like biocompatibility, long-term stability, and scalability still impede their clinical use. With ongoing advancements in biomaterials and bioengineering, stimuli-responsive hydrogels are positioned to revolutionize wound management [50].

Wound healing is a complex and highly regulated biological process that is essential for restoring skin integrity and function after injury [51,52].

Moreover, injuries caused by adhesive patches pose a significant threat to full-thickness skin trauma, as their strong adhesion can lead to severe pain and exacerbate the injury upon removal. A work inspired by the mussel has been reported, where an antibacterial hydrogel adhesive dressing has been designed and created to treat wounds effectively. Unlike most difficult-to-remove dressings, this antibacterial adhesive hydrogel showed strong adhesion, allowing painless and non-invasive on-demand removal within 2 s. The hydrogel also demonstrated excellent protein adsorption, along with strong mechanical, antibacterial, and biocompatibility properties [53,54]

Compared to traditional hydrogels, which mainly serve as passive materials for moisture retention or drug release, stimuli-responsive hydrogels can undergo reversible or irreversible physical and chemical changes in response to specific environmental cues such as pH, temperature, ROS, enzymes, or light [55]. These systems provide dynamic, microenvironment-adaptive behaviors that are especially useful in wound healing applications. The microenvironment of chronic and infected wounds is highly variable, with fluctuating pH, oxidative stress, enzymatic activity, and bacterial presence. Stimuli-responsive hydrogels are particularly well-suited to these conditions, allowing localized controlled therapeutic release, real-time responsiveness, and integration with biosensing or regenerative cues.

Stimulus-response hydrogels can react to one or multiple stimuli. For instance, changes in pH can influence inflammation, cell proliferation, bacterial growth, and oxidative stress during wound healing, thereby directly affecting the speed and quality of healing [56]. Usually, the skin maintains a slightly acidic pH between 5 and 6.5, which supports the skin barrier and inhibits harmful microorganisms. However, when the skin is injured, the pH of the wound environment changes, impacting the healing process [57,58]. Acute wounds usually have a pH between 6.0 and 6.5, while chronic wounds tend to become more alkaline, with pH levels above 7.3 [59,60]. pH-responsive hydrogels are emerging as promising materials for controlled drug release and monitoring healing progress [61]. On the other hand, photoresponsive hydrogels can alter their physical and chemical properties in response to specific wavelengths of light [62]. Such hydrogels usually contain light-sensitive functional groups or nanomaterials, allowing them to respond to ultraviolet (UV), visible (Vis), or NIR light [63]. Phototherapy mainly includes photodynamic therapy (PDT) and photothermal therapy (PTT), both of which use light to stimulate cellular processes. This approach helps prevent the development of multidrug-resistant bacteria caused by excessive antibiotic use.

PDT uses low-intensity visible light (VL) or NIR light to activate a photosensitizer, producing cytotoxic substances that kill target cells. Ag nanoparticles were incorporated into a photoresponsive metal–organic framework (MOF) hydrogel, enabling the photocatalytic generation of ROS under VL [64].

Beyond individual applications of PDT and PTT, recent studies have focused on their synergistic combination to improve therapeutic results. A composite sprayable hydrogel was designed by Liu et al. [65,66] featuring a seamlessly integrated Bi/MoS2 nano-heterojunction. By leveraging the synergistic effects of PDT and PTT, just 10 min of 808 nm NIR irradiation produced strong antibacterial effects. This accelerated both in vitro and in vivo bacterial elimination, leading to faster wound healing. In this sense, it is essential to highlight the potential applications of these materials in biomedicine, particularly in the context of the rise of infections caused by bacteria resistant to conventional antimicrobial therapies.

The rise of multidrug-resistant (MDR) bacteria is one of the most pressing global health threats of the 21st century, significantly reducing the efficacy of current antimicrobial treatments. In the U.S., nearly 23,000 people die each year from antibiotic-resistant infections [67]. As resistance mechanisms continue to accumulate in bacteria, the number of effective therapeutic options steadily declines, underscoring the urgent need for alternative strategies. In this context, novel biomedical materials are being explored to complement conventional antibiotics and reduce MDR-related morbidity and mortality [68]. Among these alternatives, photodynamic therapy (PDT) offers the advantage of localized activation through light-based devices [69]. PDT combines photosensitizers (PSs), oxygen, and light to generate reactive oxygen species (ROS), inducing non-specific photo-oxidative stress that inactivates bacteria. PSs are inert chemical molecules that can be simple dyes, such as methylene blue [70] or complex compounds such as 5-aminolevulinic acid (ALA) [71,72]. Some have demonstrated in vitro antimicrobial activity against *S. aureus*, using aloe-emodin [73] or complexed to nanoparticles for controlled release [74]. Other authors have used aerogel to apply antimicrobial photodynamic patches [75]. Additionally, CuTCPP nanosheets complexed with porphyrin-based MOF have recently demonstrated PDT activity in treating cancer, infected wounds, and periodontitis [76,77]. Photoactivatable antimicrobial patches represent, then, a promising therapeutic/preventive strategy, particularly for superficial infections and wound management. These systems leverage ROS locally, leading to broad-spectrum microbial inactivation without relying on conventional antibiotics [78]. Importantly, the mechanism of action, based on oxidative damage, is non-specific and does not target a single bacterial molecular pathway, thereby minimizing the risk of resistance development [79,80]. Additionally, the spatial and temporal control afforded by light activation enables localized treatment, reducing systemic exposure and preserving host microbiota [81]. These features make photodynamically active patches especially suitable for the management of cutaneous wounds, surgical sites, and superficial skin infections, where targeted antimicrobial activity is both feasible and desirable.

*Staphylococcus aureus*, a Gram-positive bacterium, is a primary etiological agent of superficial skin infections and has exhibited increasing resistance to multiple antibiotics, including the emergence of methicillin-resistant strains (MRSA). Approximately 30% of healthcare-associated infections (HAIs) are produced by MDR *S*. *aureus* strains [82,83,84,85]. *S. aureus* MRSA is a significant cause of HAIs such as surgical wound infections, urinary tract infections (UTIs), skin infections, and pneumonia [86,87,88,89]. Due to its clinical relevance and well-characterized pathogenicity, *S. aureus* serves as an appropriate and robust model organism for evaluating the efficacy of antimicrobial wound dressings and photoactivated therapeutic patches.

In this context, this work aims to contribute to the design and preparation of thermally stable, solvent-resistant, photoactive honeycomb films for optical processing in biological applications, based on molecular and macromolecular components, and subsequently to evaluate the utility of photoactive polymers in photodynamic therapy (PDT). This work describes the design, synthesis, characterization, and application of photoactive copolymer hydrogels derived from 2-hydroxyethylmethacrylate (HEMA) with acrylamide (AAm) monomers and the organic photosensitive compound 3,3-dimethylindoline-6′-nitrobenzospiropyran (***BSP***). The photoactive hydrogel films were initially created using 2-hydroxy-4′-(2-hydroxyethoxy)-2-methylpropiofenone as a photoinitiator and N′N-methylene bisacrylamide (MBA) at different molar percentages as a cross-linking agent, along with 3,3-dimethylindoline-6′-nitrobenzospiropyran (***BSP***), via free radical polymerization in an aqueous solution. The polymer structure was designed based on a main chain with hydrophilic functional groups, which were functionalized through the incorporation of ***BSP***, allowing these materials to adapt to potential patch applications. Finally, we focused on determining its ability to inhibit the growth of methicillin-sensitive *S. aureus* (MSSA) as well as an MDR strain, the methicillin-resistant *S. aureus* (MRSA), both of which are the primary cause of superficial infections. They were subsequently used in an assay to verify their antimicrobial properties in photodynamic therapy (PDT). This work introduces three significant contributions: (1) the functional groups of the hydrophilic hydrogel were used for functionalization with the photoactive, photochromic agent ***BSP***. The polymer’s functional groups may enable the incorporation of ***BSP*** molecules; (2) the photoactive hydrogel films were prepared using a photoinitiator, with an equimolar ratio of monomers, cross-linking agent, and ***BSP*** in aqueous solution; and (3) the photoactive hydrogel films were successfully designed for optical processing, and their photoactivity was tested in photodynamic therapy (PDT). These hydrogel films were successfully tested with the photosensitizer Ru(bpy)_2_^2+^ against *Staphylococcus aureus*.

To our knowledge, the mixture of PS Ru(bpy)_2_^2+^ with Spiropyran-like molecules has been used, but for applications other than photodynamic bacterial inactivation [90,91,92].

In this context, our strategy is novel. The mechanism of action is probably based mainly on the potential synergistic effect between ***BSP*** and Ru(bpy)_2_^2+^, and the photochromic balance of the ***BSP*** agent, which was maintained in the functionalized copolymer. This contributes to the generation of free radicals during activation, promoting energy transfer from triplet oxygen to singlet oxygen, producing highly reactive singlet oxygen (^1^O_2_), which negatively affects bacteria.

This approach broadens the potential uses of hydrogels as adhesive materials in wet environments, especially as biomaterials [93]. Looking forward, the clinical success of composite hydrogels will depend on their ability to combine therapeutic precision with functional versatility. Systems responsive to multiple stimuli are particularly suited to addressing the complex pathophysiology of chronic or infected wounds [94,95]

The structural characterization was performed using FT-IR spectroscopy. Moreover, the thermal and mechanical properties were measured, including shear tests. Thermal analysis was conducted using thermogravimetric analysis (TGA) and differential scanning calorimetry (DSC).

## 2. Results and Discussion

### 2.1. Synthesis and Characterization

The synthesis conditions for the poly(HEMA-*co*-AAm)-***BSP*** hydrogels and results are shown in Table 1.

### 2.2. Structural Characterization by FTIR Studies of the Hydrogels

**Figure 1:**
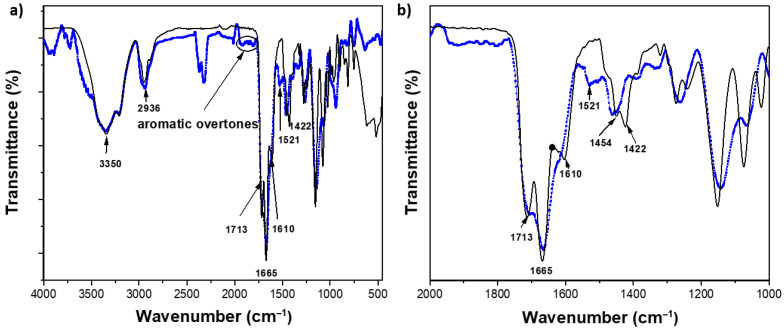
(**a**) FTIR spectrum of the copolymer and P(HEMA-*co*-AAm) with 0.5 mol.-% BSP/MBA (blue line), 0.5 mol.-% MBA (black line), range 4000–500 cm^−1^. (**b**) Wavenumber range 2000–1000 cm^−1^.

Figure 1a,b show the FTIR spectra of the poly(HEMA-*co*-AAm)-***BSP*** hydrogels at a 1:1 monomer feed ratio with MBA/***BSP*** (0.1, 0.3, and 0.5 mol.%, respectively). The FTIR spectra for the copolymer and poly(HEMA-*co*-AAm)-***BSP*** reveal changes in signal intensity for the HEMA groups after functionalization with BSP, as seen in Table 2. This can be explained by alterations in the individual peaks of –OH, –NH_2_, –CONH_2_, and –NO_2_ groups. FTIR spectroscopy displayed bands in the range of 3200–3600 cm^−1^, attributed to the stretching of the hydroxyl (–OH) and –NH deformation of the amide group; at 2847–2948 cm^−1^, related to –CH stretching of methyl (–CH_3_) and methylene (–CH_2_–) groups; the band at 1713 cm^−1^ corresponds to the –C = O vibration of the ester group from HEMA; at 1665 and 1610 cm^−1^ attributed to ester groups of ***BSP***; and at 1640–1680 cm^−1^ (C = O stretching, amide). The band at 1454 and 1422 cm^−1^ is assigned to the nitro symmetric –NO_2_ from the ***BSP*** moiety, 1335 cm^−1^ (symmetric stretching of Ar-NO_2_);1510 cm^−1^ (asymmetric stretching of Ar-NO_2_); and 1930–1836 cm^−1^ (Ar-H aromatic overtones). See Figure 1.

Table 2 summarizes the leading vibration bands in the FTIR spectra for the copolymers with and without ***BSP*** functionalization. Absorption bands in the IR spectra of the carbonyl tension bands from HEMA and ester groups have different intensities, which can generally be referred to as strong (s), medium (m), weak (w), broad, and sharp.

**Figure 2:**
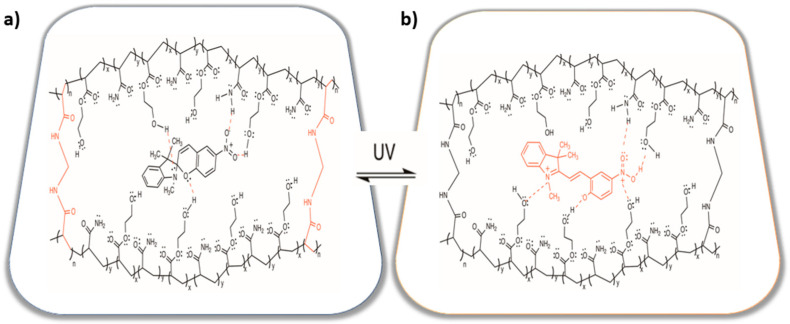
Schematic representation of the photoactive P(HEMA-*co*-AAm)-***BSP*** hydrogels, with MBA. (**a**) Before irradiation, (**b**) after irradiation, which is photoisomerized to the zwitterionic merocyanine (***MC***) form by UV light irradiation.

This variation in relative intensity of signals can be attributed mainly to hydrogen bonding interactions between the functional groups in the polymeric chains with functional ***BSP*** agent. The formation of physical attraction forces between hydrophilic functional groups (amide and hydroxyl groups) can significantly impact the intensity of variations in an infrared (IR) signal [96], see Figure 2.

### 2.3. Characterization by TGA and DSC

**Figure 3:**
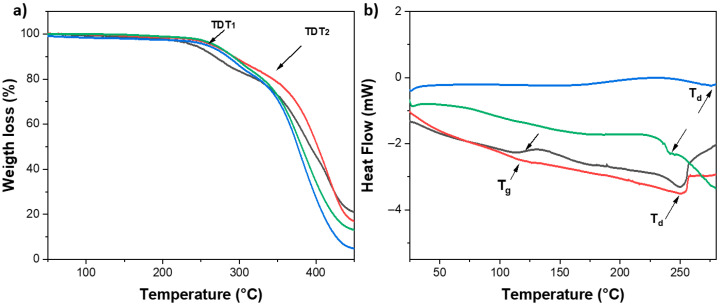
(**a**) Thermograms P(HEMA-*co*-AAm) at 1:1 feed ratio monomer composition with 0.1 mol.-% BMA (black line), 0.5 mol.-% BMA (red line), 0.1 mol.-% BMA/***BSP*** (blue line), 0.5 mol.-% BMA/***BSP*** (green line) and (**b**) DSC curves of P(HEMA-*co*-AAm) at 1:1 feed ratio monomer composition with 0.1 mol.-% BMA (black line), 0.5 mol.-% BMA (red line), 0.1 mol.-% BMA/***BSP*** (blue line), 0.5 mol.-% BMA/*BSP* (green line).

The TGA analysis of hydrogels at a 1:1 feed ratio of monomer with 0.1 and 0.5 mol.% MBA and 0.1 and 0.5 mol.% MBA/***BSP*** exhibited similar thermal decomposition, in general presented in two-step degradations, see Figure 3a. The hydrogel at 0.5 mol.-% MBA exhibited the highest thermal stability with a residual loss mass percentage of 18% for the hydrogel, with extrapolated thermal decomposition temperatures, such as TDT_1_ at 261.6 °C and TDT_2_ at 349.5 °C. The hydrogels presented similar thermal behavior but exhibited greater thermal stability for the 0.5 mol.-% MBA in the study.

Furthermore, the results obtained from the DSC curve after the 2nd heating showed the formation of amorphous polymers with pronounced glass transition temperatures (Tg) at 0.1 and 0.5 mol.-% MBA and at 0.1 and 0.5 mol.-% MBA/***BSP***. At the same time, the DSC curve of the hydrogels exhibited a second event during heating, an endothermic peak, indicating the presence of a decomposition reaction (Td) of the material, which confirms the information provided by the TGA analysis (see Figure 3b). On the other hand, the Tg value at 0.1 mol.-% MBA was slightly lower but more pronounced, indicating a greater free volume in the hydrogel cells and greater movements of the functional groups and chains. The Tg value increased at a concentration of 0.5 mol.% MBA and was less pronounced, indicating more restricted movements of the chains and functional groups due to the higher percentage of cross-linking. Tg values were found at 119.2 and 121.4 ± 0.1 °C, respectively. The results indicated that the hydrogels exhibit a higher Tg value at higher mol.% MBA than for polymer chain [97], influenced by the reticulation reaction [98,99]. On the other hand, in the hydrogels functionalized with ***BSP***, the Tg was not evident, indicating a less flexible system, that is, a more rigid one. The presence of ***BSP***, inserted between the polymer’s backbones, decreased the interaction between these latter, and hence reduced the thermal stability of the entire hydrogel matrix. Additionally, the FTIR results were supported, confirming that ***BSP*** was present in the hydrogel matrix, interacting with the polymer primarily through hydrogen bond interactions.

### 2.4. Swelling Behavior

**Figure 4:**
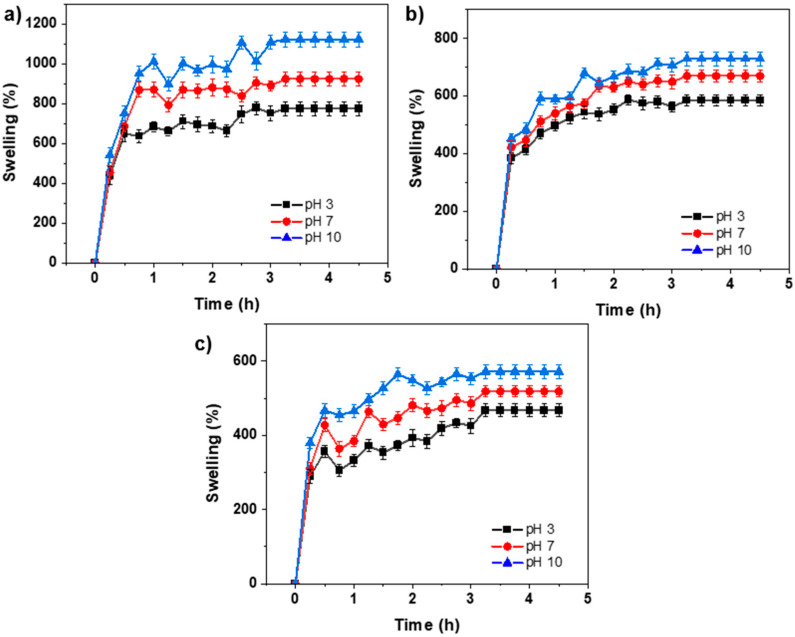
Hydration tests of the copolymer P(HEMA-*co*-AAm) 1:1 feed ratio of monomer at pH 3 (black line), pH 7 (red line), and pH 10 (blue line); (**a**) at 0.1 mol.-% MBA/***BSP***, (**b**) at 0.3 mol.-% MBA/***BSP***, (**c**) at 0.5 mol.-% MBA/***BSP***. The experiments were performed in triplicate, and the standard deviation was determined (σ < 5%).

Figure 4 shows the swelling behavior of the hydrogels at 0.1, 0.3, and 0.5 mol-% MBA/***BSP***. The hydration capacity of poly(HEMA-*co*-AAm)-***BSP*** hydrogels was examined as a function of pH (pH = 3, 7, and 10). The swelling of the hydrogels increased with pH, reaching its maximum at pH 10. This is due to the strengthening of hydrogen-bonding interactions between the carboxylate (-COO-) groups and water. Additionally, swelling increased over time but eventually stabilized, reaching a state of equilibrium. Conversely, the percentage of hydration decreased as the percentage of crosslinking agent increased at 0.5 mol-% MBA/***BSP***, as shown in Figure 4. This is likely due to the increased stiffness of the polymer matrix. It is important to note that the presence of BSP did not significantly affect the swelling behavior of gels compared to the degree of crosslinking.

### 2.5. Lap Shear Test of Polymeric Hydrogel

Lap shear strength testing measures the ability of a material to withstand stresses set in a plane, where the exerted shear force is moving the two substrates in opposite directions. The test consists of loading a specimen comprising a 3-ply laminate: substrate/polymer/substrate.

The hydrogel with feed monomer ratios of 1:1 at 0.1 mol.-% MBA displayed less stiffness but with a lower resistance to failure, see Table 3. Moreover, the hydrogel with a 1:1 feed monomer ratio at 0.5 mol.-% MBA sustained higher tension of failure shear, that is, a better shear resistance against the applied force in wet conditions as the failure shear stress increases. The mechanical properties of the hydrogels can be modulated based on the various kinds of technological applications [100,101,102,103].

These results indicate that a 1:1 feed monomer ratio with 0.5 mol.-% MBA/***BSP*** has a higher failure shear stress and shows better adhesive properties. In this study, we designed and synthesized an adhesive hydrogel that depends on dynamic interactions between functional groups and the hydrophilic substrate. Unlike most hydrogels, which can be challenging to remove, this adhesive hydrogel demonstrates soft adhesion properties, enabling gentle and non-invasive removal for potential patch applications. Table 4 presents some results obtained for biological applications, which are compared with the findings from this work.

### 2.6. Antimicrobial Properties Based on Photodynamic Therapy (PDT)

Uncontrolled bleeding and infection can significantly increase the risk of death. Hydrogel sealants have gained notable attention for their ability to control bleeding. However, because interfacial water acts as a strong barrier to solid surface bonding, the ongoing challenge is to find a product that provides strong tissue adhesion while also offering anti-infective properties.

Inspired by the adhesion mechanism of the biofilm P(HEMA-*co*-AAm) [107] in the present work, we introduce a novel adhesive hydrogel based on a synthetic P(HEMA-*co*-AAm) and the photochromic agent ***BSP***, in conjunction with the cross-linker agent MBA through a straightforward radical photopolymerization process. ***BSP*** was incorporated into the adhesive polymer to combine the properties of both the essential adhesive hydrogel and the optical component (***BSP***), exhibiting light-responsive properties under UV–Vis stimulus. P(HEMA-*co*-AAm)-***BPS*** displayed adequate adhesion, between 4.34 ± 0.91 and 6.18 ± 1.02 kPa, and excellent antibacterial properties. Therefore, P(HEMA-*co*-AAm)-***BSP*** represents a promising class of biomaterials for hemostasis and wound healing with sufficient efficiency. Table 4 presents bioadhesive patches designed for use in wet environments (hemostatic) that have shown limited adhesion to moist tissue surfaces, with an adhesive strength of less than 40 kPa [23,53,104,105,106,107,108]. An ideal bioadhesive for wet conditions should be both rigid and elastic, allowing minimal resistance to the natural deformation of dynamic soft tissues such as skin, which ranges from 0.1 to 100 kPa [109,110].

We first evaluated the antimicrobial activity of the Ru(bpy)_2_^2+^ photosensitizer to decrease the viability of *S. aureus* bacteria. A liquid culture containing 1 × 10^7^ CFU/mL of *S. aureus* (both MRSA and MSSA) was exposed to 0–8 µg/mL of Ru(bpy)_2_^2+^ and activated with 17 µW/cm^2^ of blue light at 450–460 nm for 10 min.

**Figure 5:**
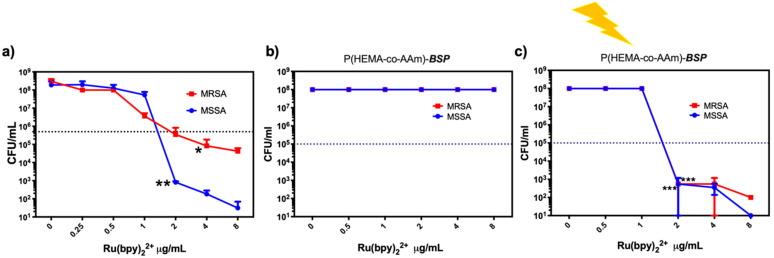
The photodynamic antimicrobial activity of the P(HEMA-*co*-AAm)-***BSP*** copolymer in combination with the Ru(bpy)_2_^2+^ photosensitizer. The photodynamic antimicrobial activity against methicillin-susceptible (MSSA) and methicillin-resistant (MRSA) *Staphylococcus aureus* was assessed: the MIC of the photosensitizer Ru(bpy) was determined between 0 and 8 µg/mL (**a**), the intrinsic antibacterial activity of P(HEMA-*co*-AAm)-***BSP*** was evaluated in the dark (**b**), and the reduction in MIC for P(HEMA-*co*-AAm)-***BSP*** and PS activated by light was assessed by embedding 0–8 µg/mL Ru(bpy) in the P(HEMA-*co*-AAm)-***BSP*** matrix (**c**). Bacterial viability is expressed as the log_10_ of the mean ± SD, with * *p* < 0.05, ** *p* < 0.01, and *** *p* < 0.001 from a two-tailed *t*-test with comparison to control bacteria in the dark.

Figure 5a shows that Ru(bpy) significantly reduces the bacterial viability of MSSA by 6 log_10_ at 2 µg/mL (*p* < 0.01). For MRSA at 2 µg/mL, bacterial viability was reduced by 3 log_10_ (*p* < 0.05). Higher concentrations of Ru(bpy)_2_^2+^ significantly reduced the bacterial viability of the MSSA strain, achieving complete inactivation at concentrations ranging from 4 to 8 µg/mL; however, this effect was not observed for the MRSA strain. The results indicated that, while free Ru(bpy)_2_^2+^ required concentrations of 4–8 µg/mL to eliminate methicillin-sensitive (MSSA) strains, partial inactivation of methicillin-resistant (MRSA) strains was achieved. The incorporation of the photoactive hydrogel films improved their efficacy, reducing the minimum inhibitory concentration (MIC) to 2 µg/mL to completely inactivate MSSA and MRSA strains. These results demonstrate that the combined effect of hydrogel films containing ***BSP*** and Ru(bpy)_2_^2+^ within a hydrogel film not only enhances antimicrobial activity but also supports the potential of these photoactive films as innovative photosensitive antimicrobial platform coatings. This synergistic effect of ***BSP*** and Ru(bpy)_2_^2+^ suggests that these materials are promising candidates for next-generation antimicrobial coatings and as creative photosensitive materials. The antimicrobial activity of the developed materials shows promising results.

To preserve the photodynamic properties of the Ru(bpy)_2_^2+^ photosensitizer when embedded in the photoactive P(HEMA-coAAm)-***BSP*** hydrogel, the matrix was polymerized at the bottom of 24-well plates. The photosensitizer in aqueous solution was then applied as a thin layer over the photoactive hydrogel matrix at concentrations ranging from 0 to 8 µg/mL. The layer was left for 30 min to allow the Ru(bpy)_2_^2+^ photosensitizer to embed into the photoactive hydrogel matrix. S. aureus strains MRSA and MSSA were inoculated onto the matrix at 1 × 10^7^ CFU/mL, incubated in the dark for 10 min, and samples were collected. Next, the plate was illuminated with blue light at 17 µW/cm^2^ at 450–460 nm for 10 min to activate the PS, and the samples were then collected again. As shown in Figure 5b, the photoactive hydrogel film with Ru(bpy)_2_^2+^, which was not exposed to light, showed no antimicrobial activity compared to the control. Conversely, the irradiated plate showed increased antimicrobial activity, as seen in Figure 5c, achieving complete inactivation of MSSA and MRSA with 2 µg/mL of Ru(bpy)_2_^2+^, reducing the bacterial viability by 7 log_10_. This rise in photodynamic activity may be due to the hydrogel matrix’s optical properties.

**Figure 6:**
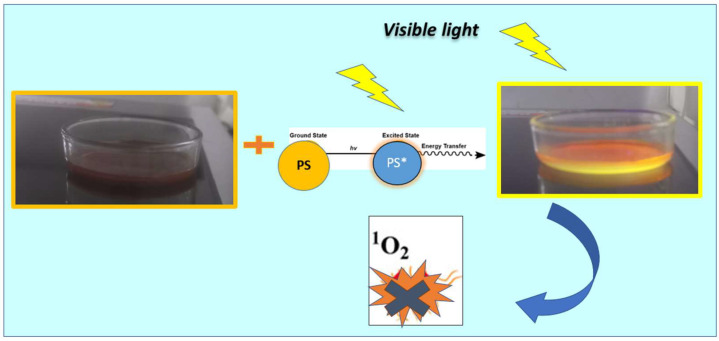
The schematic representation of the photoactive hydrogel-BSP combined with Ru(bpy)_2_^2+^, as the photosensitizer, in photodynamic antimicrobial activity.

The mechanism of action is probably based on especially the potential synergistic effect between ***BSP*** and Ru(bpy)_2_^2+^ and the photochromic balance of the BSP agent, which was maintained in the functionalized copolymer, as shown in Figure 6. The photochromic agent and the functionalized copolymers form an isomeric structure called merocyanin (***MC***), which has a conjugated π bond in the conjugated system, with a significant resonance effect induced by a nitro group capable of absorbing energy and being excited in the visible light region. This occurs due to the decrease in energy through the electronic transition of the double bond system from π to π*, where the excited state is more polar than the ground state, making radiation mostly effective, resulting in a significant bathochromic shift in the visible range and a hypochromic effect of lower intensity. Furthermore, the electronic transition π* from a basal singlet state to an excited singlet state is very likely to happen, since the spin of the excited electron is unpaired with its antiparallel spin, and its relaxation mechanism is fast and reversible [111]. On the other hand, PDT requires light irradiation of a specific wavelength to excite the ***BPS*** moiety. The ***BPS*** moiety in its lowest energy level (ground singlet state, π) is shifted to the short-lived excited singlet state (1π*), which can be converted into the long-lived excited triplet state (3π*). In the presence of ambient oxygen, the triplet state can undergo two types of reaction mechanisms: (I) the transfer of electrons to form toxic reactive oxygen species (such as peroxide, H_2_O_2_, hydroxyl radicals, etc.) and (II) a mechanism involving energy transfer to ground-state triplet oxygen to produce highly reactive singlet oxygen (^1^O_2_). Both reactions can coincide. However, not all highly conjugated unsaturated organic molecules can undergo intersystem crossing to produce the triplet state necessary for photochemical reactions. This contributes to the generation of free radicals during activation, promoting energy transfer from triplet oxygen to singlet oxygen, producing highly reactive singlet oxygen (^1^O_2_), which negatively affects bacteria [112,113].

## 3. Conclusions

Photoactive films were successfully designed for optical processing, and their photoactivity was evaluated in photodynamic therapy (PDT). On the other hand, the photoactive hydrogel films have good mechanical strength, thermal stability, and soft adhesive properties.

The photoactive hydrogel film, based on poly(2-hydroxyethylmethacrylate-*co*-acrylamide), was functionalized with ***BSP*** through physical interactions via a polymerization reaction. The optically active ***BSP*** is incorporated into the hydrogel, which is linked through physical interactions via the functional groups of the polymer chain. These macromolecular components lead to the formation of a solid film that, when combined with a photosensitizer, facilitates its use in sanitary pads, hydrogels, or dressings that aid in treating or preventing superficial infections where light can easily activate both components, ***BSP*** and Ru(bpy)_2_^2+^ photosensitizer. The light-emitting properties of the polymer films were characterized by the emission of blue and violet colors when exposed to UV light.

The results indicated that, while free Ru(bpy)_2_^2+^ required concentrations of 4–8 µg/mL to eliminate methicillin-sensitive (MSSA) strains, partial inactivation of methicillin-resistant (MRSA) strains was achieved. The incorporation of BSP into the hydrogel films improved their efficacy, reducing the minimum inhibitory concentration (MIC) to 2 µg/mL to completely inactivate MSSA and MRSA strains. These results demonstrate that the combined effect of hydrogel films containing ***BSP*** and Ru(bpy)_2_^2+^ within a hydrogel film not only enhances antimicrobial activity but also supports the potential of these photoactive films as innovative photosensitive antimicrobial platform coatings. This synergistic effect of ***BSP*** and Ru(bpy)_2_^2+^ suggests that these materials are promising candidates for next-generation antimicrobial coatings and as creative photosensitive materials. The antimicrobial activity of the developed materials shows promising results.

The mechanism of action is based mainly on the potential synergistic effect between ***BSP*** and Ru(bpy)_2_^2+^ and the photochromic balance of the ***BSP*** agent, which was maintained in the functionalized copolymer. This contributes to the generation of free radicals during activation, promoting energy transfer from triplet oxygen to singlet oxygen, producing highly reactive singlet oxygen (^1^O_2_), which negatively affects bacteria.

This approach broadens the potential uses of hydrogels as adhesive materials in wet environments, especially as biomaterials. Looking forward, the clinical success of composite hydrogels will depend on their ability to combine therapeutic precision with functional versatility. Systems responsive to multiple stimuli are particularly suited to addressing the complex pathophysiology of chronic or infected wounds.

## 4. Materials and Methods

### 4.1. Materials

Chemicals were used without further purification and were of analytical grade. Acrylamide (AAm, Sigma-Aldrich, St. Louis, MO, USA) and 2-hydroxyethyl methacrylate (HEMA, 99% Merck, Darmstadt, Germany) were used as the monomers. Benzoyl peroxide (BPO, 99.98%, Sigma-Aldrich, St. Louis, MO, USA); 1,3,3-Trimethyl-2-methylen-indoline (97%, Sigma-Aldrich, St. Louis, MO, USA); 2-hydroxy-5-nitrobenzaldehyde (98% Sigma-Aldrich), 2,3,3-trimethylindoline, 2-bromoethanol, 2-butanone, trimethylamine, 2-bromoethanol (C_2_H_5_BrO, Sigma-Aldrich, St. Louis, MO, USA), and 2-butanone (C_2_H_5_COCH_3_, 99.0%, Sigma-Aldrich, St. Louis, MO, USA) all were commercially obtained from Sigma-Aldrich (St. Louis, MO, USA). 2-Hydroxy-4′-(2-hydroxyethoxy)-2-methylpropiofenone (IRGACURE 2959, 99.5%, Merck, Darmstadt, Germany) was used as a photoinitiator, and all analytical-grade reagents were of high purity. *N*′*N*-methylene bisacrylamide (MBA, 99.5% Merck, Darmstadt, Germany) was used as a cross-linking agent. All chemicals were used as received without further purification.

### 4.2. Analytical Techniques

The hydrogel samples’ IR spectra were recorded at room temperature through Perkin Elmer (USA). Fourier transform infrared spectroscopy (FTIR) spectra were acquired by a Spectra 2000 using the attenuated total reflection ATR–FTIR method, in the range of 400 to 4000 cm^−1^. The TGA measurements were performed at a heating rate of 10 °C/min under a nitrogen atmosphere with a flow rate of 150 cm^3^/min using a thermogravimetric analyzer (TGA/DSC1 1100 SF, Mettler Toledo, Barcelona, Spain). The hydrogel sample size (between 3–4 ± 0.1 mg) was used in each experiment. Thermal analysis by differential scanning calorimetry (DSC) was performed in a nitrogen atmosphere using a DSC Mettler Toledo 822e analyzer. The measurements were carried out at a heating rate of 10 °C/min under a nitrogen atmosphere with a flow rate of 50 cm^3^ min^−1^. All experiments were measured from 25 °C to 550 °C in an inert atmosphere (N_2_ gas).

### 4.3. Synthesis of 3,3-Dimethylindoline-6′-Nitrobenzospiropyran (BSP)

**Figure 7:**
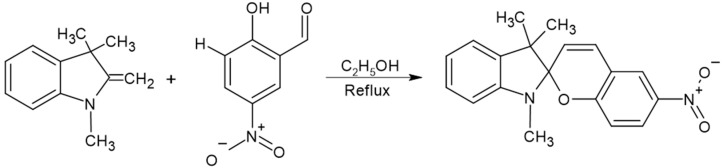
Reaction scheme for the synthesis of trimethylindoline-6-nitro-spirobenzopyran (***BSP***).

The synthesis was conducted in a 250 mL three-neck flask placed in an oil bath equipped with a magnetic stirrer and a condenser. To the flask, 4 mL (23 mmol) of 1,3,3-trimethyl-2-methylene-indoline and 4 g (23 mmol) of 2-hydroxy-5-nitrobenzaldehyde were added in 80 mL of ethanol. The mixture was heated until boiling at 78 °C for 5 h. Upon completion of the reaction, a dark green solid product was obtained, which was filtered and dried on filter paper in an oven for 72 h. The product obtained from the synthesis was purified by crystallization. Solubility tests were conducted using various solvents, including chloroform, ethanol, methanol, and acetone, which indicated that a 1:1 mixture of methanol and acetone showed improved solubility at elevated temperatures. In an Erlenmeyer flask equipped with a heating mantle, 50 mL of the 1:1 methanol and acetone solvent mixture was heated to boiling. The resulting product (solute) was then added, and the solvent mixture was gradually introduced in small amounts until the solid was completely dissolved. The Erlenmeyer flask was wrapped in absorbent paper to allow for slow cooling of the solution. It was covered with a watch glass to facilitate crystallization, which took place over 96 h. After this time, vacuum filtration was performed, followed by cold methanol washes. The final product obtained was a dark yellow or faintly brown crystalline solid, as shown in Figure 7.

### 4.4. Preparation of Hydrogel Films

The hydrogel films were prepared by free radical polymerization using an equimolar monomer feed ratio 1:1 (HEMA/AAm), in the presence of 2-hydroxy-4′-(2-hydroxyethoxy)-2-methylpropiophenone. (IRGACURE 2959) as photoinitiator, and MBA as cross-linker agent, and 1,3,3-trimethyl-2-methylene-indoline (***BSP***) as photochromic agent, based on a procedure previously reported by our group [23]. The polymerization reaction was carried out in aqueous solution using irradiation. A general method is described for a 1:1 feed ratio monomer HEMA/AAm. Briefly, 15 mmol of each monomer (50:50 mol-%), 0.5 mmol-% of IRGACURE 2959, MBA, and ***BSP*** (at 0.1 and 0.5 mmol-%) were dissolved in 8 mL of distilled water, and a 250 µL aliquot was added into sterile 24-well cell culture plates. Subsequently, the mixture was exposed to UV radiation for 3 h. The Poly(HEMA-*co*-AAm)-***BSP*** hydrogels were maintained sealed to maintain their hydration state. Finally, the dried hydrogels were characterized by Fourier Transform Infrared (FTIR) spectroscopy, thermal properties, swelling behavior, and mechanical properties.

### 4.5. Swelling Studies

The swelling properties of polymers were investigated to develop absorbent materials with suitable swelling characteristics for bio-applications.

The initial weight of the hydrogel samples was accurately measured after being freeze-dried. The experiments were performed in triplicate, and the standard deviation was determined (σ < 5%).

Then, the samples were immersed in a citrate buffer (pH < 7) or phosphate buffer (pH > 7) at 25 °C and were swollen until the equilibrium state was reached. In this way, the dried samples of the copolymers were placed in a solution with a defined pH (3, 7, and 10). After specified time intervals, the swollen hydrogels were weighed after gently removing excess water using filter paper. The swelling ratio is defined using the following equation (Equation (1)):

(1) $$DS(\%)=\frac{\left(W_{S}-W_{d}\right)}{W_{d}}\times 100$$

where *W_s_* is the weight of the swollen hydrogel at an equilibrium state, and *W_d_* is the weight of the dried hydrogel (Xerogel).

### 4.6. Lap Shear Test

The objective of the shear test is to subject samples of the material to a state of shear stress. For this purpose, two portions of hydrogel are positioned between two cavities formed by three sandwiched plates of the assembly accessory. In an idealized way, each sample adopts a parallelepiped geometry with dimensions h = 1 mm, a = 5 mm, and b = 5 mm. Then, the assembly is rested in ambient conditions for 24 h and later tested in a Cellscale 5000 biaxial tensile testing machine, equipped with a 10 N load cell, through movement relative between the plates in the longitudinal direction of the assembly. The shear stress in the reference configuration is calculated as $\tau =\frac{V}{A}$, where V is the shear force in one of the samples, which, given the double shear configuration, can be calculated as $V=\frac{F}{2}$, while A corresponds to the initial cut area $A=ab=25\;mm^{2}$. Furthermore, the tangential deformation is calculated as $\gamma =\frac{\Delta }{h},$ where Δ corresponds to the relative displacement between jaws during the test. The adhesive strength was determined from the maximal loading over the area of the adhesive overlap. For the repeated adhesion tests, a waiting time of 10 min was needed before the next lap-shear cycle [100].

### 4.7. Determination of Antimicrobial Photodynamic Properties

The methicillin-sensitive MSSA and methicillin-resistant MRSA strains of Staphylococcus aureus were used in the assays. Bacteria were cultured in either solid or liquid Trypticase soy medium, as needed. In liquid medium, bacteria were grown to an optical density of OD600 nm = 0.2–0.4. For photodynamic therapy (PDT), bacteria were adjusted to 1 × 10^7^ CFU/mL in a phosphate-buffered saline (PBS) solution. The 1 × 10^7^ UFC/mL bacteria were placed in the wells of a 24-well plate over P(HEMA-*co*-AAm)-BSP hydrogels, with the photosensitizer Ru(bpy)_2_^2+^ [114] at various concentrations. Illumination was performed in a light box with 17 mW/cm^2^ for 10 min using a blue LED lamp (450–460 nm), delivering 61.2 J/cm^2^. After illumination, bacterial viability was assessed by broth microdilution and colony counting on plates after 16–20 h of incubation in Mueller–Hinton medium, as previously described [115]. Bacterial viability was reported as the mean ± SD in CFU/mL. Each assay was conducted in technical triplicate within three independent biological replicates, resulting in a total of n = 9 per data point. The MIC of the photosensitizer was determined by mixing 1 × 10^7^ CFU/mL bacteria with an increasing concentration of Ru(bpy)_2_^2+^, ranging from 0 to 8 μg/mL.

### 4.8. Statistical Analysis

For PDT, statistical analyses were performed using Prism 9.0 Software (GraphPad Software, LLC, San Diego, CA, USA). For parametric data, significance was assessed with two-tailed *t*-tests and one-way ANOVA for lethality curves, followed by Tukey’s post-test. Bacterial viability was assessed after PDT exposure and expressed as log_10_ of mean ± SD with * *p* < 0.05, ** *p* < 0.01, and *** *p* < 0.001 from a two-tailed *t*-test with comparison to control bacteria in the dark.

## Figures and Tables

**Table 1:** Experimental conditions for the synthesis of photoactive poly(HEMA-*co*-AAm)-***BSP*** at 1:1 monomer ratio and at various concentrations of MBA and ***BSP*** (0.1, 0.3, and 0.5 mol.%).

Feed Monomer Ratio	[AAm] (mol/g)	[HEMA] (mol/g)	[MBA] (mol.-%)	[*BSP*] (mol.-%)	Yield (%)
P(HEMA-*co*-AAm) 1:1	0.015/1.065	0.015/1.950	0.0	0.0	95
P(HEMA-*co*-AAm) 1:1	0.015/1.065	0.015/1.950	0.1	0.0	90
P(HEMA-*co*-AAm) 1:1	0.015/1.065	0.015/1.950	0.3	0.0	90
P(HEMA-*co*-AAm) 1:1	0.015/1.065	0.015/1.950	0.5	0.0	92
P(HEMA-*co*-AAm) 1:1	0.015/1.065	0.015/1.950	0.1	0.1	90
P(HEMA-*co*-AAm) 1:1	0.015/1.065	0.015/1.950	0.3	0.3	90
P(HEMA-*co*-AAm) 1:1	0.015/1.065	0.015/1.950	0.5	0.5	90

Accordingly, poly(HEMA-*co*-AAm) and copolymer-***BSP*** hydrogels always yielded higher than 90%.

**Table 2:** Main vibration bands of the *BSP*-functionalized copolymers.

Hydrogel	AAm	HEMA	Ester	-NO_2_
	(C = O)	(C = O)	(C = O)	-N-O
P(HEMA-*co*-AAm) with 0.1 mol.% MBA	1658	1713 (s)	(w)	---
P(HEMA-*co*-AAm) with 0.1 mol.% MBA/BSP	1659	(w)	1613 (s)	1454 (s)
P(HEMA-*co*-AAm) with 0.5 mol.% MBA	1656	1713 (s)	(w)	---
P(HEMA-*co*-AAm) with 0.5 mol.% MBA/BSP	1657	(w)	1610 (s)	1454 (s)

**Table 3:** Mechanical properties of the poly(HEMA-*co*-AAm) and photoactive poly(HEMA-*co*-AAm)-***BSP*** hydrogels at a 1:1 monomer feed ratio.

Feed Monomer Ratio	MBA (mol.-%)	*BSP* (mol.-%)	Shear Stress (kPa)	Shear Modulus (kPa)
P(HEMA-*co*-AAm) without BSP	0.1 0.5	0.0 0.0	0.67 ± 033 1.97 ± 0.25	1.69 ± 0.46 3.33 ± 1.27
P(HEMA-*co*-AAm) with MBA and BSP	0.1 0.5	0.1 0.5	1.81 ± 0.37 3.41 ± 0.17	4.34 ± 0.91 6.18 ± 1.02

The experiments were performed in triplicate, and the standard deviation was determined (σ < 5%).

**Table 4:** Adhesive hydrogel biofilms and antibacterial abilities.

Polymeric Materials	Biomedical Application	Young’s Modulus (E) *	Reference
Hydroxyethyl methacrylate –co-acrylamide with Dopamine	Catechol-based bioinspired adhesive properties in a wet medium	33.37 ± 2.74 − 58.86 ± 2.04 kPa	[23]
Maleic anhydride-modified β-cyclodextrin (CD), amantadine as a competitive guest	Adhesive antibacterial hydrogel	37.4 − 85.8 37.6 kPa	[53]
Chitosan grafted with methacrylate, dopamine, and N-hydroxymethyl acrylamide	Biofilm-inspired adhesive antibacterial hydrogel	34.0 kPa	[104]
Carboxymethyl chitosan hydrogel	Biodegradable carbohydrate polymers	2.3 kPa to 13.3 kPa, 12.5 kPa	[105]
Allyl cellulose with Dopamine	Mussel-inspired cellulose-based adhesive hydrogel	38.8 to 40.2 kPa	[106]
Hydroxyethyl methacrylate –co-acrylamide with cross-linker (MBA)	Biomimetic adhesive hydrogel	1.39 ± 0.06 kPa, 3.85 ± 1.87 kPa	[107]
Hydroxyethyl methacrylate with acrylamide, *N*′*N*-methylene bis-acrylamide (MBA) and photochromic agent (***BSP***)	Photoactive hydrogels as materials for biological applications, such as antimicrobial patches	4.34 ± 0.91 6.18 ± 1.02 kPa	This work

* Tensile and compressive testing.

## Data Availability

The data presented in this study are available on request from the corresponding author.
